# Supplementary material for: Global prioritization schemes vary in their impact on the placement of protected areas
Source: PLoS One. 2025 Jan 2;20(1):e0307730. doi: 10.1371/journal.pone.0307730 (PMC11695003; doi:10.1371/journal.pone.0307730)
Supplement: S1 File — (DOCX) [file pone.0307730.s001.docx]

# Supporting Information

**Global prioritization schemes vary in their impact on the placement of protected areas**

Tjaden-McClement et al.

**Supplemental Methods**

*World Database on Protected Areas*

We included WDPA protected areas that had a reported area but were represented as spatial points (rather than polygons) by creating circular buffers around each point with their reported areas, constraining buffers within the borders of the country of origin. We filtered the dataset to include only 100% terrestrial PAs and those with “Designated”, “Inscribed”, or “Established” status. The WDPA was further processed to create yearly datasets of protected area to date from 1980 to 2021, with any overlapping PAs removed to ensure accurate measures of protection over time.

**Table**. Summary of matching covariates used to select spatial grid cells as control matches for grid cells in the Biodiversity Hotspots and Last of the wild prioritization schemes. and their rationales for inclusion, time frame, and data sources.

| **Matching Covariate** | **Rationale** | **Time frame** | **Source** |
| --- | --- | --- | --- |
| Human footprint | Used as inclusion criteria for Biodiversity Hotspots (high human footprint)^1^ and Last of the Wild (low human footprint)^2^ | 1995 – 2004 | [Global Human Footprint, v2](https://sedac.ciesin.columbia.edu/data/set/wildareas-v2-human-footprint-geographic) |
| Agricultural potential | Affects likelihood of protection^3^ | 2007 | Global agriculture opportunity cost ([Naidoo & Iwamura, 2007](https://doi.org/10.1016/j.biocon.2007.07.025)) |
| Elevation | Affects likelihood of protection^3^ | 2010 | [Global Multi-resolution Terrain Elevation Data - GMTED2010](https://topotools.cr.usgs.gov/gmted_viewer/gmted2010_global_grids.php) |
| Population density | Affects likelihood of protection^3*^ | 2000 | [GRUMP: population Density grid v1](https://sedac.ciesin.columbia.edu/data/set/grump-v1-population-density) |
| Road Density | Affects likelihood of protection^3^ | Circa 2000 | Roads layer from [Data from: Global terrestrial Human Footprint maps for 1993 and 2009](https://datadryad.org/stash/dataset/doi:10.5061/dryad.052q5) |
| Biome | Ensures matches come from ecologically similar areas | 2001 | [WWF Terrestrial Ecoregions of the World (TEOW)](https://www.worldwildlife.org/publications/terrestrial-ecoregions-of-the-world) |
| Country | Ensures matches come from geographically and politically similar areas | Current | E.g., [Administrative boundaries of countries of the world](https://databasin.org/datasets/e00ff45d152346658890f3e0f127b7b1/) |

^1^ Myers, N., Mittermeier, R. A., Mittermeier, C. G., Fonseca, G. A. B., & Kent, J. (2000). Biodiversity hotspots for conservation priorities. Nature, 403(February), 853–858.

^2^ Sanderson, E. W., Jaiteh, M., Levy, M. A., Redford, K. H., Wannebo, A. V., & Woolmer, G. (2002). The human footprint and the last of the wild. BioScience, 52(10), 891–904. doi:10.1641/0006-3568(2002)052[0891:THFATL]2.0.CO;2

^3^ Joppa, L. N., & Pfaff, A. (2009). High and far: Biases in the location of protected areas. PLoS ONE, 4(12), 1–6. doi:10.1371/journal.pone.0008273

^*^ Joppa & Pfaff (2009) found a bias for protected areas being location at father distances from cities, rather than areas of lower population density.


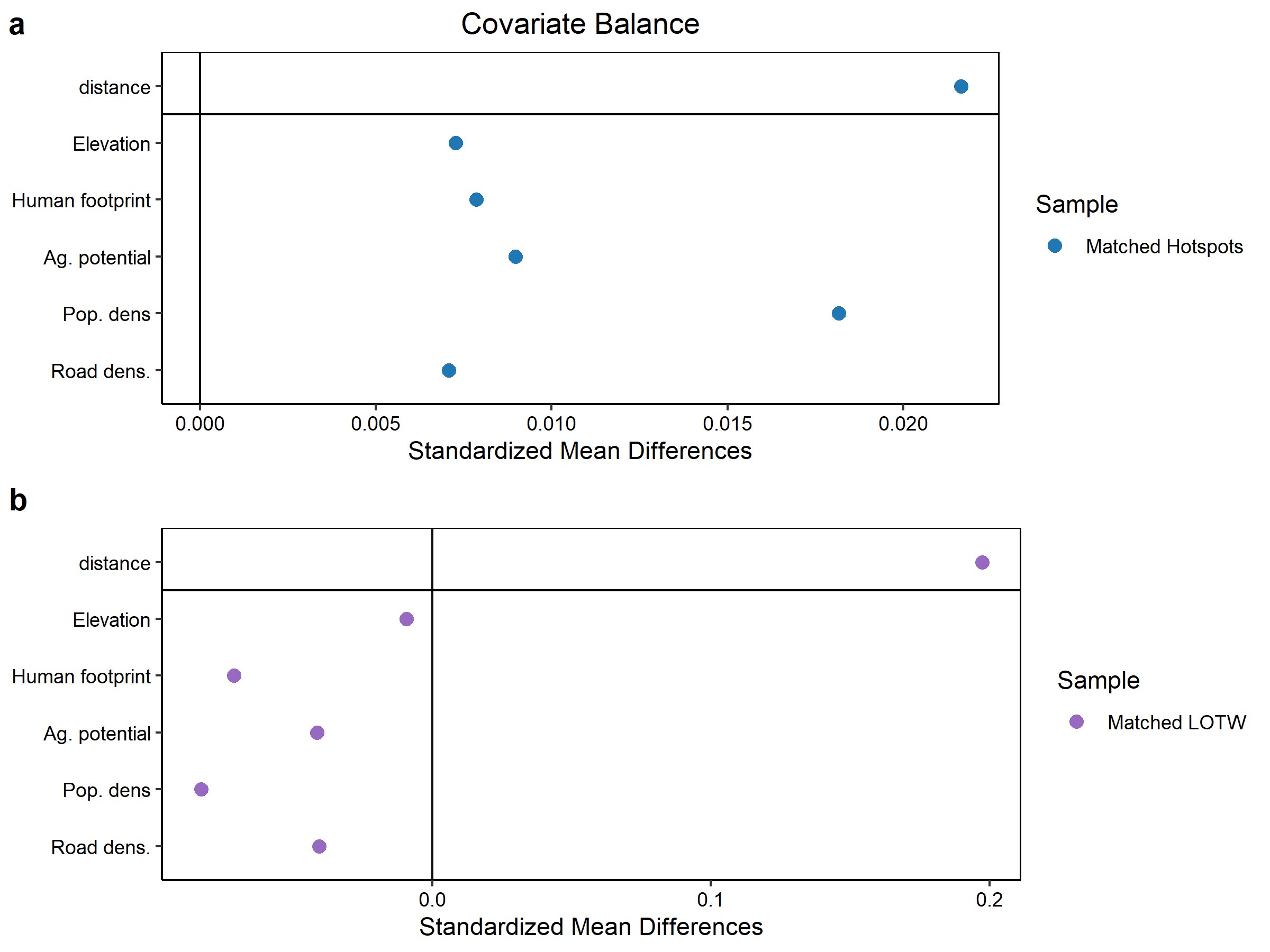


**Fig A.** Love plots showing the standardized mean differences in the 5 matching covariates (elevation, agricultural potential, human footprint, population density, and road density) and overall distance between a) Biodiversity Hotspots and b) Last of the Wild samples and their matched controls.

**
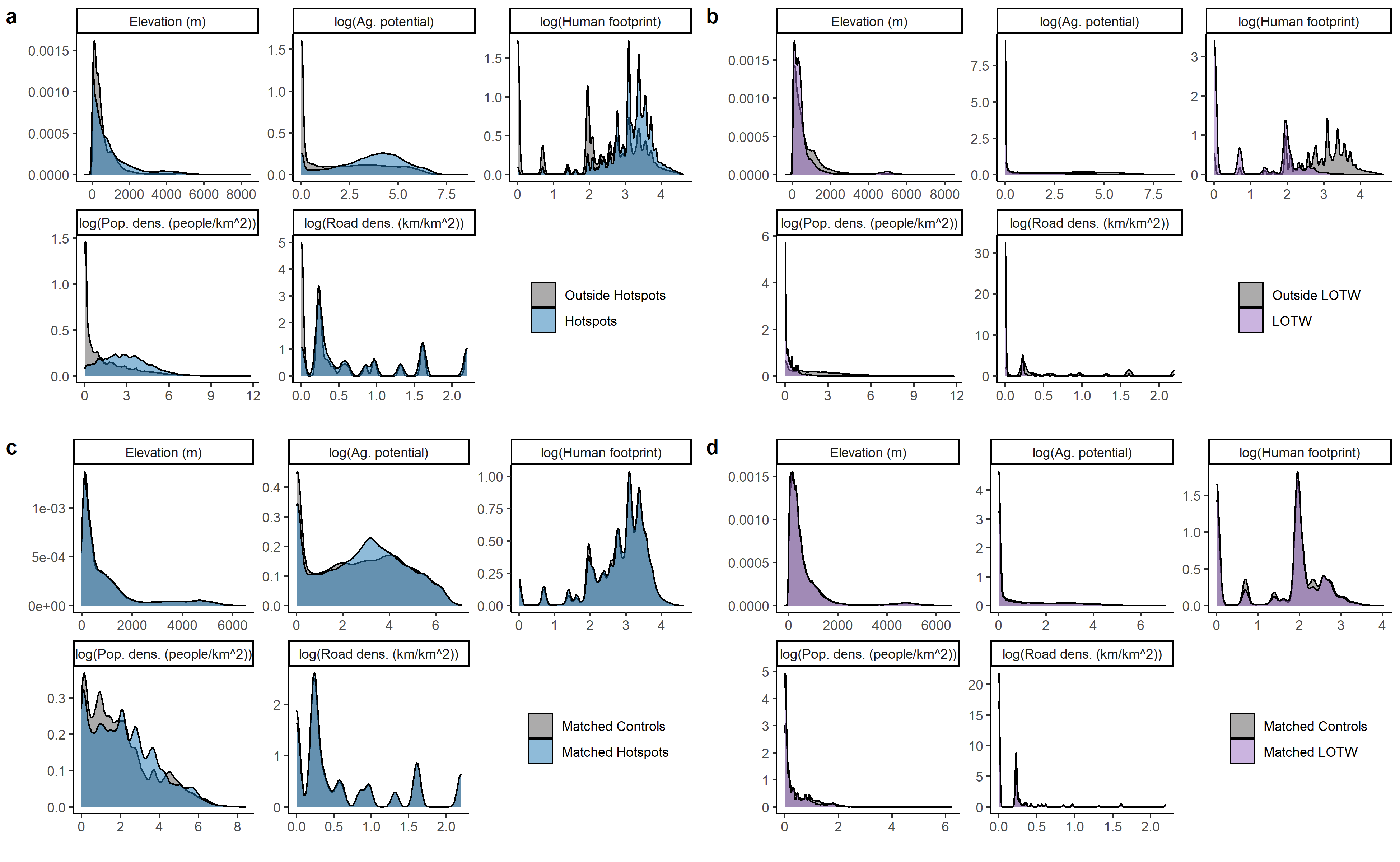
Fig B**. Distribution of matching covariates (elevation, agricultural potential, human footprint, population density, and road density) inside vs. outside of the Biodiversity Hotspots and Last of the Wild prioritization schemes before matching (a, b) compared to after matching in the selected control on priority groups (c, d). Agricultural potential, human footprint, population density, and road density were log transformed for visualization purposes, with a constant of 1 added to avoid negative values due to 0’s and values < 1.
