# Supplementary material for: Global prioritization schemes vary in their impact on the placement of protected areas
Source: PLoS One. 2025 Jan 2;20(1):e0307730. doi: 10.1371/journal.pone.0307730 (PMC11695003; doi:10.1371/journal.pone.0307730)
Supplement: Attachment — Submitted filename: PA_Prioritization_PLOS_One_Response to Reviewers.docx [file pone.0307730.s002.docx]

**Response to Reviewers**

Manuscript title: Global prioritization schemes vary in their impact on the placement of protected areas

Original Submission ID:  PONE-D-24-28142

*Dear Dr. Gaglio,*

*Thank you for recognizing the merit in our work and providing feedback in support of a revised manuscript. We also thank the reviewers for their detailed reviews.*

*Below we provide point-by-point responses to the editor and reviewer comments (our responses in blue italics with original comments in* normal black font*). We have made the corresponding revisions in a tracked changes version of the manuscript (provided along with a clean version of the revised manuscript). Line numbers provided in the responses refer to the revised manuscript with tracked changes.*

*In response to reviewers’ comments, we have expanded on the implementation process for protected areas and associated costs, updated number formatting, and organized our discussion with subheadings (in addition to the other responses below).*

*We hope that you agree with our responses and revisions, and that you find the revised manuscript suitable for publication in* PLOS One.

Sincerely,

Cole Burton (on behalf of all co-authors)

3. … Figures 1 and 2 in your submission contain [map/satellite] images which may be copyrighted. All PLOS content is published under the Creative Commons Attribution License (CC BY 4.0), which means that the manuscript, images, and Supporting Information files will be freely available online, and any third party is permitted to access, download, copy, distribute, and use these materials in any way, even commercially, with proper attribution. For these reasons, we cannot publish previously copyrighted maps or satellite images created using proprietary data, such as Google software (Google Maps, Street View, and Earth). For more information, see our copyright guidelines: http://journals.plos.org/plosone/s/licenses-and-copyright.

*Our Figures 1 and 2 do not contain copyrighted images. Figure 1 shows maps that we created to depict the areas designated as priorities by the Biodiversity Hotspots and Last of the Wild prioritization schemes. The underlying spatial data for these schemes was accessed from publicly available datasets (credited in our manuscript), and the country boundaries used as the basemap are public domain (Natural Earth, https://www.naturalearthdata.com/). Figure 2 shows the “priority” and “control” points used in our analysis for each scheme, which we generated using the statistical matching method described in the manuscript (and again uses the public domain basemap of countries). Maps for both figures were created by us (authors) in R. To the best of our knowledge, no copyright permissions are required for these figures.*

**Review Comments to the Author**

Reviewer #1: Overall, the quality of this paper is good.

*Thank you for your positive feedback.*

There should be a conclusion section.

*The concluding paragraph is now specifically denoted by a Conclusion sub-heading.*

The contributions of the paper should be expand and further highlighted. For example, there are many other matching studies associated with protected areas' conservation status. How does this study differ from existing studies.

*We added new text and citations (lines 101, 126-129) to highlight how our study is unique and differs from previous studies that have used matching and causal inference techniques to examine other aspects of protected area effectiveness. This addition complements our text and citations highlighting previous studies on protected area coverage (e.g. Maxwell/Venter/Butchart, lines 80-87) and emphasizing how our study takes a novel approach by specifically assessing the impact of prioritization schemes using a causal inference approach (lines 99-104, 121-135)*

The discussion can be restructured to highlight the points being discussed, such as a seperate sub-section of research limitation; a sub-section of suggestions for further research; a sub-section of policy implications.

*We added sub-sections for “Research Limitations and Future Directions” and “Conclusion” (and policy implications are intertwined within several sections).*

Reviewer #2: In this study, the authors conduct a much-needed assessment of the effectiveness of two conservation prioritisation schemes, i.e., LOTW and BHs, when protecting biodiversity throughout key priority areas. Overall, this study is scientifically sound and exceptionally well-written. I would like to extend my thanks to the authorship team for their dedication to an important research topic. I only have relatively minor comments for the authors to consider, but by no means expect them to include them all in a revised manuscript.

*Thank you for your thoughtful review and helpful suggestions.*

Specifically, the role of PAs in generating ecotourism opportunities and thus income for local, rural or indigenous communities has not been mentioned in the introduction, I suggest including a sentence or two on this to provide some context on the wider benefits of PAs beyond just conserving biodiversity.

*We added a sentence in the introduction to provide context for the wider benefits of PAs, as suggested (lines 45-47).*

Can the authors expand on the implementation process of PAs in relation to their economic costs, as this concept has been touched on in the abstract and discussion but not adequately explained in enough detail for readers unfamiliar with the process to understand, i.e., what makes a particular site more/or less costly to protect?

*We provided more information about the potential costs associated with establishing a PA, which are typically higher in regions with more human settlement/development (lines 71-77). We believe this revision now more effectively frames the citations that follow regarding biases in protected area placement.*

Please see below for more detailed comments.

**Detailed comments:**

Title – while I am not against titles featuring questions, I think that this title could be improved by specifically stating the main takeaway message here. Something along the lines of “Last of the Wild protected areas, but not Biodiversity Hotspots, are effective at protecting global biodiversity in key priority areas”.

*We appreciate this suggestion and have opted for a compromise (“Global prioritization schemes vary in their impact on the placement of protected areas”), which we feel better conveys the key result while remaining succinct and of general interest. (And we note that the specific priority schemes are included as Keywords and in the Abstract.)*

Line 33 – the term “cheaper” here is a little confusing without relevant clarification. Either expand on what you mean here or use alternative phrasing.

*This referred to the lower costs of creating a protected area in areas without competing human uses (e.g. housing, agriculture). We updated the phrasing to make this clearer (lines 34-35).*

Line 53 – E.O. Wilson’s book Half-Earth springs to mind here.

*While this book is relevant to the wider discussion of calls to protect nature, it provides fewer specific targets for how and when to achieve the goal of protecting 50% of global area for nature, so we cited the similar Nature Needs Half movement.*

Line 56 – management also includes commitment to protect and enforcement/prosecution by bad actors by regional authorities, too. Perhaps expand on this a little.

*We added a citation “Di Minin, E., & Toivonen, T. (2015). Global protected area expansion: creating more than paper parks. BioScience, 65(7), 637-638.” (line 60) which emphasizes the importance of management, but we save further elaboration on this point for the discussion in order to keep the introduction focused on PA placement and representativeness.*

Line 132 – what spatial resolution were the PA data downloaded in?

*The World Database of Protected Areas provides PA data as vector data (polygons and points) with the precise location of PAs, so there is no spatial resolution associated with the PA data.*

Line 132 – why did the authors decide on 5 x 5 km grid cells? What was the justification for this? I ask because I have the IUCN Red List Area of Occupancy 2 x 2 km resolution in mind… understand that this could be due to computing power, etc. Perhaps some extra clarification on the decision-making process here is needed.

*The 5 x 5 km spatial scale used was a compromise between precision and computational efficiency. This scale allowed us to compute spatial analyses within several days, and at a global scale still provides meaningful insight into patterns in PA coverage. We have added a sentence to this effect to clarify this decision (lines 148-150).*

Table 1 – not sure whether scientific notation for 1.00e+00 is necessary here?

*We removed the scientific notation for the two instances of “1.00e+00” in Table 1.*

Line 250 – again reconsider the use of scientific notation in text here, I don’t see the issue with writing out the value in full here?

*We used the full values as suggested (line 265).*

Table 2 – P value column, why not just present very small values as “< 0.0001”? same for model estimates (SE).

*We updated Table 2 as suggested, and removed scientific notation as suggested above.*

Line 335 – avoid contractions, i.e., “did not”.

*We removed this contraction (line 349) and those identified below (lines 360, 392).*

Line 336 – such as what? Can you provide some examples of unobserved variables for context here?

*We added “number of threatened species” as an example of an unobserved variable for illustration (lines 350-351).*

Line 346 – contraction.

Line 359 – I agree.

Line 368 – reconsider the word “contemporaneously” …

*We changed the phrasing to “developed in the same time frame” (line 382-3).*

Line 376 – contraction.

Reviewer #3: One of the most importat conservation actions challenges on this days are the prioritization for decision makers. This papper present another way to approch this question.

This manuscript is technically clear, also the statistical analysis is decribed and can be replicable and all data is available.

*Thank you for the positive feedback.*

- Lines 78 to 82 is in the introduction part but without references, goes better on the discussion part.

*We believe that this theorized mechanism of how a prioritization scheme would translate into placement of new protected areas is relevant in the introduction of the analysis to give the reader context for the causal link between schemes and our proposed outcome.*

- Lines 313 to 318

313 It is likely that certain Hotspots received greater protection or other conservation

314 investment than non-priority sites, particularly by Conservation International (CI), the

315 organization that developed this prioritization scheme. The Critical Ecosystem Partnership Fund

316 (CEPF), of which CI is a partner, has invested about US$150 million in biodiversity conservation

317 projects in Hotspots since 2001 (CEPF, 2021). However, our results suggest that this investment

318 did not impact the rate of formally protecting Hotspots as a whole, relative to control areas.

Please explain better this, seems like is direct correlate the financial investment to the results, but it can be other factors.

*This section was intended to highlight that there have been significant investments made for conservation in Biodiversity Hotspots, and while this is valuable, the rate of protection was not still not higher than control areas. There was not a positive correlation between the financial investment and our results. We have rephrased the final sentence to improve clarity (lines 330-332).*

- Line 344 reference Joppa & Pfaff 2008, its not listed on references.

*This referred to Joppa & Pfaff 2009, we have corrected the error (Line 358)*

- Line 354 reference Mittermier et al, with no year I assume that is MitermEier et al 2004, please correct.

*We added the year (2004; line 369).*

- Line 462 reference Robert J. Hijmans; other references are first the last name and only the initials of the name.

*We updated the reference (line 490).*
